# OTX-202 Smartphone App to Reduce Suicidal Ideation Among High-Risk Transition-Age Youth: Open-Label, Single-Arm, Phase 1 Clinical Trial

**DOI:** 10.2196/89248

**Published:** 2026-05-01

**Authors:** Craig J Bryan, Jarrod Hay, Noah Treangen, Lauren R Khazem

**Affiliations:** 1 Larner College of Medicine University of Vermont Burlington, VT United States; 2 College of Medicine The Ohio State University Columbus, OH United States

**Keywords:** suicide, cognitive behavioral therapy, mobile health, mHealth, mobile app, smartphone, OTX-202

## Abstract

**Background:**

The transition from adolescence to adulthood (18 to 25 years) is associated with an increased risk of suicidal ideation and behaviors. Suicide-focused cognitive behavioral therapies (CBTs) have been shown to significantly reduce suicidal ideation and behaviors but are not widely available to high-risk individuals. Digital therapeutics could improve access to these treatments.

**Objective:**

This study aimed to evaluate the acceptability, safety, and potential efficacy of OTX-202 among transition-age youth (18 to 25 years) receiving mental health care outside an inpatient hospital setting.

**Methods:**

In this phase 1 single-arm clinical trial, 59 transition-age youth with recent suicidal ideation or suicide attempts used OTX-202, a smartphone app designed to deliver suicide-focused CBT, concurrently with usual outpatient mental health care. After baseline, eligible patients completed 12 weekly assessments of suicidal ideation, depression, and anxiety.

**Results:**

From baseline to week 12, participants reported statistically significant, large reductions in suicidal ideation (mean difference –5.1, 95% CI –6.5 to –3.7; *d*=0.95). In total, 3 (5.1%; 95% CI 0%-11.2%) participants reported suicide attempts. Reductions in suicidal ideation and suicide attempt rates were consistent with results from previously published randomized clinical trials of suicide-focused CBTs. Participants rated OTX-202 in the 97th percentile of usability and completed a mean of 9.0 (SD 3.5) of 12 app modules, supporting the app’s acceptability. There were no patient deaths, device-related events, or severe adverse events, supporting the app’s safety.

**Conclusions:**

Results support the safety, acceptability, and potential efficacy of OTX-202 for reducing suicide risk among transition-age youth.

**Trial Registration:**

ClinicalTrials.gov NCT06008132; https://clinicaltrials.gov/study/NCT06008132

## Introduction

Globally, more than 700,000 people die by suicide each year, with 1 in every 100 deaths worldwide resulting from suicide [1]. Although global suicide rates have generally declined, variability across nations is evident. In the United States, suicide rates increased by more than 33% from 1999 to 2023, with some of the largest and fastest increases occurring among children and young adults [2]. Suicide rates increase markedly during the transition from late adolescence to early adulthood; in 2023, for instance, the suicide rate was 2.3 per 100,000 among individuals aged 10 to 14 years, 9.8 per 100,000 among those aged 15 to 19 years, and 17.3 per 100,000 among those aged 20 to 24 years [2]. The relative increase in suicide rates during this transition period could be related to experiencing multiple significant developmental milestones, including leaving school, entering the workforce, establishing independent living, and forming new relationships. This transition period is also associated with high rates of suicidal ideation, an important risk factor for suicide [3]. More than 12% of individuals aged 18 to 25 years report experiencing suicidal ideation annually, nearly double the rate of older adult age groups [4].

Multiple psychotherapies have been shown to reliably reduce suicidal ideation, although larger effects are generally seen with psychotherapies that directly target suicidal ideation [5], also known as *suicide-focused psychotherapies*. Of the various suicide-focused psychotherapies that have been shown to significantly reduce suicidal ideation, suicide-focused cognitive behavioral therapies (CBTs) have shown some of the largest average effects [5]. Unlike other suicide-focused therapies, suicide-focused CBTs have also been shown to reliably reduce suicide attempts [5], possibly because rapid reductions in suicidal ideation are associated with subsequent reductions in the risk of suicide attempts [6-9].

Despite their efficacy, suicide-focused CBTs and other suicide-focused interventions have not been widely adopted by mental health professionals [10], are often implemented with poor quality or in combination with contraindicated clinical practices [11-13], and are underemphasized relative to other recommended suicide prevention practices [14]. Therefore, individuals with suicidal behaviors who access mental health care are unlikely to receive the most effective treatments. Digital therapeutics, defined as patient-facing software applications designed to help patients prevent, manage, or treat medical conditions [15], could address these issues. Digital therapeutics may be especially useful for reducing suicidal ideation among transition-age youth, who generally view digital therapeutics as a possible tool for avoiding stigmatization while affording more customizable experiences than traditional mental health care delivery models [16]. The OTX-202 app, a smartphone-based digital therapeutic designed to deliver suicide-focused CBT, is one promising candidate.

Results of a recent randomized clinical trial of OTX-202 showed sustained reductions in suicidal ideation relative to an active comparator among psychiatric inpatients hospitalized for elevated suicide risk [17]. Among patients with a lifetime history of suicide attempts, OTX-202 also resulted in significant reductions in postdischarge suicide attempt rates. However, because patients in that study were recruited during inpatient psychiatric hospital stays for acutely elevated suicide risk, the results may not generalize to patients receiving mental health treatment outside a hospital setting. In this study, we aimed to evaluate the acceptability, safety, and potential efficacy of OTX-202 among transition-age youth (aged 18 to 25 years) receiving mental health care outside an inpatient hospital setting. We hypothesized that OTX-202 would demonstrate acceptability and be associated with reductions in suicidal ideation and psychological symptoms.

## Methods

### Study Design

This study was an open-label, single-arm, phase 1 clinical trial conducted at The Ohio State University, a large, urban, academic medical center and tertiary referral hospital serving a diverse patient population across the Midwestern United States.

### Participants

Participants were transition-age youth who (1) were aged 18 to 25 years, (2) reported a suicide attempt within the past month and/or suicidal ideation with intent to die within the past week, (3) owned a smartphone capable of downloading and running OTX-202 and other data collection apps, (4) were able to complete enrollment and informed consent procedures, (5) were currently receiving mental health treatment, and (6) were able and willing to provide at least 2 verifiable contacts for emergency purposes. Individuals were excluded if they (1) had untreated or unmanaged psychosis, (2) were unable to consent due to intoxication, (3) were unable to consent due to cognitive impairment, or (4) had a medical condition that could compromise or otherwise impair their ability to complete the study or their safety.

### Procedures

Interested individuals were referred to the study team by their mental health treatment providers or self-referred after viewing digital recruitment advertisements posted on Instagram. Interested individuals first completed informed consent procedures. After providing consent, participants completed an initial screening and eligibility assessment. Eligible individuals then completed a baseline assessment. At the end of the baseline assessment visit, researchers helped participants download the OTX-202 app onto their smartphones, complete onboarding and setup steps, and receive a tutorial on app use. For the first 2 weeks, participants received 4 prompts per day at pseudorandom times between 8 AM and 10 PM to answer ecological momentary assessment (EMA) items. Each EMA survey took <5 minutes to complete. For the first 12 weeks, participants also met with a researcher weekly to complete follow-up assessments, track adverse events (AEs) and device-related problems, and monitor suicide risk. Weekly assessments took <30 minutes to complete. All assessments were completed remotely using internet-based videoconferencing platforms.

### Intervention

Participants were required to be actively engaged in mental health treatment throughout the duration of the study. Participants were not restricted from meeting with their preferred mental health treatment professional or receiving any recommended mental health treatments while participating in this research study and were encouraged to inform their mental health providers about their participation in the study. All concomitant medications and psychotherapeutic interventions received by participants were recorded at baseline and at each weekly follow-up assessment.

In addition to usual care, all participants downloaded and had access to the OTX-202 app, also named Aviva. The OTX-202 app includes 12 educational modules drawn from suicide-focused CBT protocols that have been shown to significantly reduce suicide attempts [18-23]. Each module takes approximately 10 to 15 minutes to complete. OTX-202 helps participants identify personal warning signs for a suicidal crisis, use self-management and distraction strategies, and identify sources of social support, as well as provides one-touch access to the National Suicide Prevention Hotline and emergency services. These strategies can be updated and/or changed over time. In addition to these steps, the app includes a series of modules and video vignettes designed to introduce and teach additional self-management strategies that can reduce emotional distress and avert suicidal crises (eg, relaxation, mindfulness, and cognitive worksheets). Finally, the OTX-202 app also helps participants develop a schedule for practicing skills and reporting their progress to their mental health clinician. A chatbot guides users through each module. Explanatory animated videos explain new ideas to users and why they are important for reducing suicide risk. The app also includes clinical vignette videos in which individuals discuss their personal experiences with each session, demonstrating the relevance and utility of the app. Skill use throughout the week is reinforced by interactive widgets that prompt practice. The app is compatible with iOS and Android smartphone operating systems.

### Outcomes

The primary outcome was suicidal ideation, assessed using 2 complementary methods. The first method assessed momentary suicidal ideation during the first 2 weeks of intervention use from baseline to day 14 via EMA. Each EMA prompt included items from the Self-Injurious Thoughts and Behaviors Interview–Revised (SITBI-R) [24] that assess suicidal thoughts, urges, and plans. Participants were asked to report the intensity of each item “right now,” ranging from 0 (not at all) to 4 (extremely). Item responses were summed to provide an overall metric of suicidal ideation severity, with higher scores reflecting more severe suicidal ideation (range 0-44). The second method assessed past-week suicidal ideation from baseline to week 12 with the Scale for Suicide Ideation (SSI) [25]. The SSI assesses the intensity of suicidal thoughts, urges, and behaviors. Item responses are summed, with higher scores indicating more severe suicidal ideation (range 0-38).

Secondary outcomes included suicide attempts, assessed with the SITBI-R [24], and psychological symptom severity, assessed with the depression and anxiety subscales of the Patient-Reported Outcomes Measurement Information System (PROMIS) [26]. The SITBI-R distinguishes suicide attempts (including aborted, interrupted, and actual attempts) from preparatory behaviors and nonsuicidal self-injury using a yes-or-no response format. PROMIS depression and anxiety subscale item responses are summed and converted into standardized T scores with a mean of 50 and SD of 10, with higher scores indicating more severe symptoms.

App acceptability and usability were assessed using normalized scores from the System Usability Scale (SUS) [27] and user engagement. The SUS assesses user perceptions of effectiveness, efficiency, and satisfaction, with higher scores indicating more positive perceptions (range 0-100), and scores >68 reflecting above-average usability relative to other devices and systems [28,29]. User engagement was defined as the number and percentage of app modules completed.

### Safety Monitoring

AEs, device-related events, and unanticipated problems were monitored weekly with the Systematic Assessment for Treatment Emergent Effects–Systematic Inquiry [30].

### Ethical Considerations

The study protocol was reviewed by the US Food and Drug Administration (FDA) to obtain an investigational device exemption (#G220117) and by The Ohio State University Biomedical Institutional Review Board (protocol #2022H0217) and was preregistered on August 15, 2023, at ClinicalTrials.gov (NCT06008132). To facilitate safety monitoring, the FDA required that all participants be actively engaged in mental health treatment while enrolled in the study, with prespecified safety reviews by an independent safety monitor when 20 and 40 participants were enrolled. Informed consent was obtained in person or remotely from all individuals participating in the study using a videoconferencing platform and electronic consent procedures compliant with US federal requirements (21 Code of Federal Regulations part 11) [31]. To protect participant privacy and confidentiality, data were collected and stored using numeric participant IDs in electronic databases meeting Health Insurance Portability and Accountability Act (HIPAA) storage requirements. This report follows the CONSORT (Consolidated Standards of Reporting Trials) reporting guidelines for eHealth interventions [32]. Participants received US $1 for each completed EMA survey and US $20 for each completed weekly assessment.

### Statistical Analysis Plan

To assess change in momentary suicidal ideation, we used mixed-effects models with a fixed effect for week, a random slope specified at the day level, and a random intercept specified at the participant level to account for repeated EMA scores per day and individual differences within and between participants. To assess change in SSI, PROMIS, and SUS scores, we constructed separate mixed-effects models for each outcome with week entered as a fixed-effect predictor and random intercepts and slopes specified at the participant level. For all models, covariance matrices were selected via comparison of Bayesian information criterion values, with smaller values indicating better fit [33]. To assess the clinical significance of our findings, we supplemented the primary analyses with calculations of within-group effect sizes (Cohen *d*), the percentage of patients who denied suicidal ideation (scores of 0 on SSI items 4 and 5), and the percentage of patients with PROMIS depression and anxiety scores within normal limits of the general population (T score ≤55). Suicide attempts, AEs, and module completion were summarized using descriptive statistics (eg, frequencies and percentages).

A priori power analyses were conducted using a web-based app designed to estimate power for EMA studies [34] and the GLIMMPSE (General Linear Mixed Model Power and Sample Size) online sample size software [35] for mixed-effects modeling. The EMA protocol included 4 EMA prompts per day for 14 consecutive days. A minimum sample size of 60 provided >80% power to detect large effects with 50% missing data. The SSI, PROMIS, and SUS were assessed weekly for 13 consecutive weeks. A minimum sample size of 60 provided 95% power to detect a small (Cohen *f* >0.10) effect of time with a 2-tailed α=.05 and a moderate correlation among repeated measures (*r*=0.50). Regarding missing data, 1 (1.7%) participant was withdrawn after providing consent because they became ineligible upon reaching their 26th birthday. Therefore, data were available from only 59 participants. Of these, data were available from 1540 of 3304 (46.6%) possible EMA prompts and 718 of 767 (93.6%) possible weekly assessments. Analyses included all available data from these participants, with missing data handled using maximum likelihood estimation, and were conducted using SAS (version 9.4; SAS Institute Inc) supplemented with SPSS (version 29.0; IBM Corp).

## Results

### Participant Characteristics

**Figure 1:**
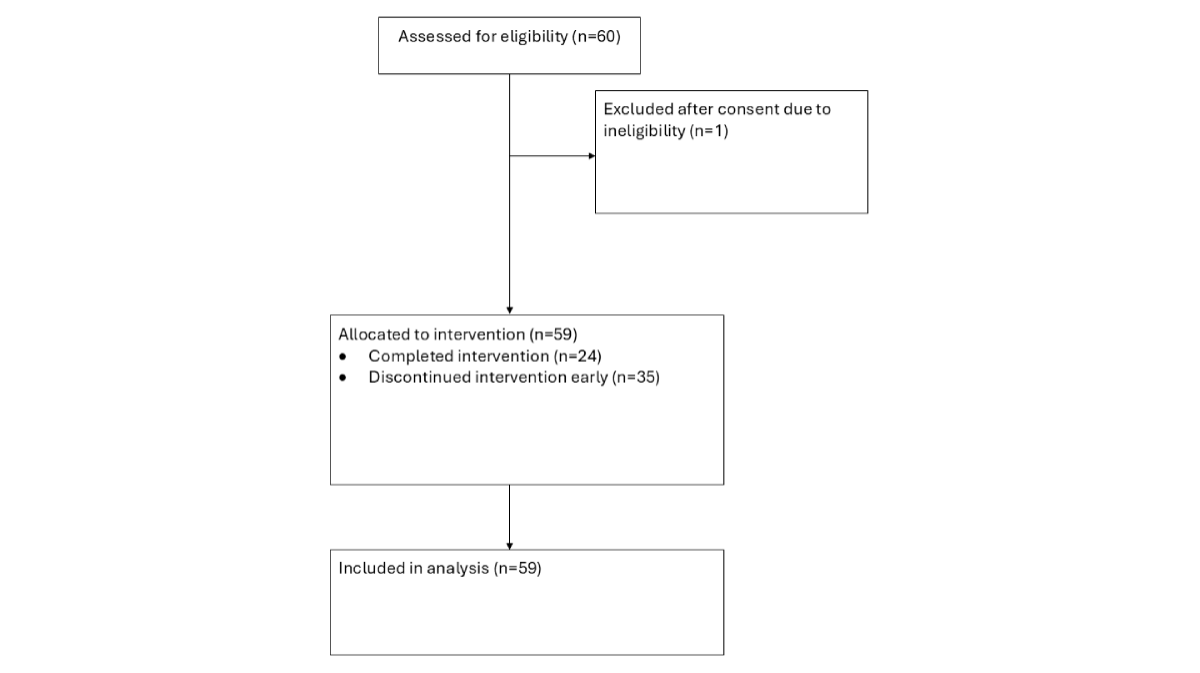
Flowchart of the participants enrolled in this study.

Of 60 individuals screened for eligibility, 59 were enrolled in the study and exposed to the intervention, and 1 was enrolled but subsequently removed from the trial prior to intervention exposure because they turned 26 years old, rendering them ineligible (Figure 1). Sample characteristics are reported in Table 1. The mean age of the participants was 21.4 (SD 2.1; range 18-25) years; 53 (89.8%) self-reported being biologically female and 6 (10.2%) being biologically male; 42 (71.2%) self-identified as woman, 9 (15.3%) as man, and 8 (13.6%) as nonbinary; 28 (63.3%) self-identified as Caucasian, 10 (16.7%) as Asian, and 8 (13.3%) as African American or Black; and 22 (37.3%) self-identified as straight or heterosexual, 15 (25.4%) as bisexual, and 15 (25.4%) as something else. Most participants (n=48, 81.4%) reported participating in psychotherapy and taking psychotropic medications. At baseline, 52 (88.1%) participants reported suicidal ideation within the past week on the SSI, 45 (76.3%) reported prior nonsuicidal self-injury, 43 (72.9%) reported prior preparatory behavior, 38 (64.4%) reported prior aborted attempts, 17 (28.8%) reported prior interrupted attempts, and 31 (52.5%) reported prior actual attempts. Mean symptom scores at baseline were as follows: SSI, 10.3 (SD 5.2); PROMIS depression, 63.7 (SD 6.8); and PROMIS anxiety, 66.3 (SD 7.8).

**Table 1:** Sample characteristics (N=59).

Characteristics		Participants
Age (years), mean (SD)		21.4 (2.1)
**Biological sex, n (%)**		
	Male	6 (10.2)
	Female	53 (89.8)
**Gender identity, n (%)**		
	Men	9 (15.2)
	Women	42 (71.2)
	Nonbinary	8 (13.6)
**Hispanic or Latinx ethnicity, n (%)**		
	No	53 (89.8)
	Yes	6 (10.2)
**Race, n (%)**		
	African American or Black	8 (13.6)
	Asian	10 (16.9)
	Caucasian or White	38 (64.4)
	Native Hawaiian or other Pacific Islander	2 (3.4)
	Other	1 (1.7)
**Sexual orientation, n (%)**		
	Gay or lesbian	6 (10.2)
	Straight or heterosexual	22 (37.3)
	Bisexual	15 (25.4)
	Something else	15 (25.4)
	Refused	1 (1.7)
**History of self-injurious behaviors, n (%)**		
	Nonsuicidal self-injury	45 (76.3)
	Preparatory behavior	43 (72.9)
	Aborted attempt	38 (64.4)
	Interrupted attempt	17 (28.8)
	Actual attempt	31 (52.5)
**Psychiatric diagnoses^a^, n (%)**		
	Depressive disorder	51 (86.4)
	Anxiety disorder	42 (71.2)
	Trauma- or stressor-related disorder	19 (32.2)
	Neurodevelopmental disorder	10 (16.9)
	Obsessive-compulsive or related disorder	7 (11.9)
	Feeding and eating disorder	6 (10.2)
	Personality disorder	6 (10.2)
	Bipolar or related disorder	4 (6.8)
	Substance-related or addictive disorder	2 (3.4)
	Schizophrenia or other psychotic disorder	1 (1.7)
	Dissociative disorder	1 (1.7)
	Sleep-wake disorder	1 (1.7)
	Disruptive, impulse-control, or conduct disorder	1 (1.7)
**Concomitant treatment, n (%)**		
	Psychotropic medication only	3 (5.1)
	Psychotherapy only	8 (13.6)
	Psychotherapy and medication	48 (81.4)

^a^Self-reported by participant.

### Change in Suicidal Ideation

Mean EMA-assessed suicidal ideation scores significantly decreased from week 1 (mean 9.1; 95% CI 7.6-10.6) to week 2 (mean 8.3; 95% CI 6.7-9.9): mean difference −0.8, 95% CI −1.5 to −0.002; *t*_253_=2.0; *P*=.049; *d*=0.26.

**Figure 2:**
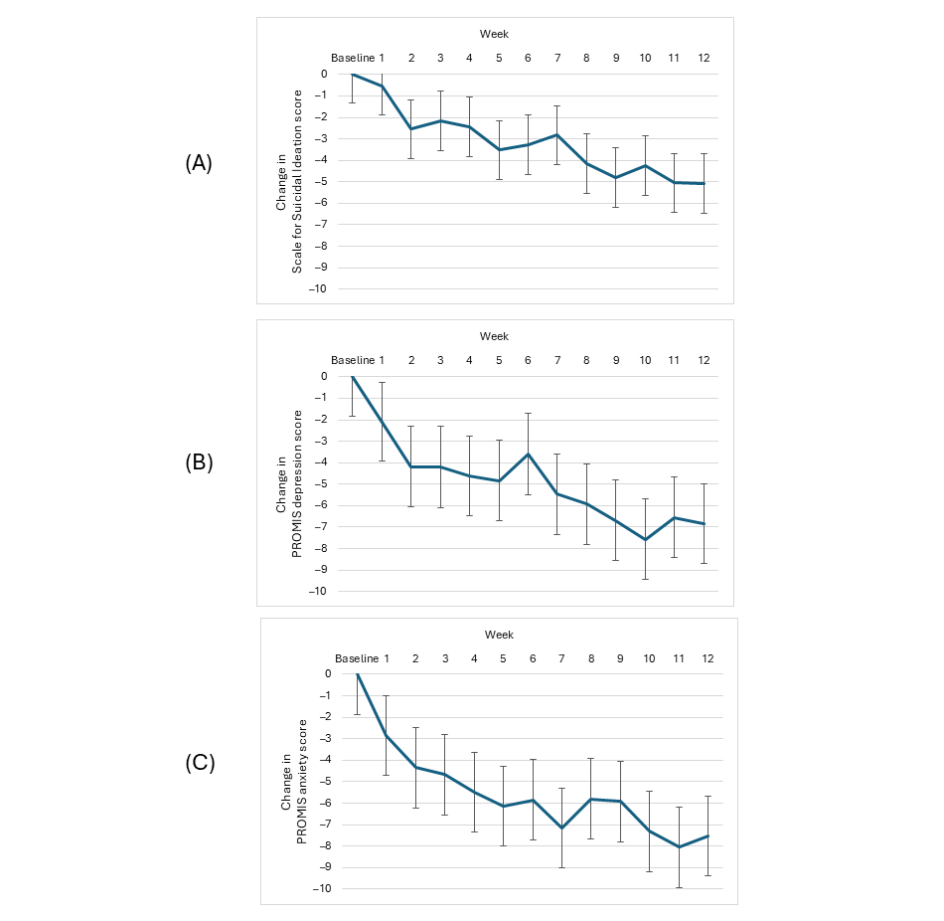
Postbaseline change in weekly (A) suicidal ideation, (B) depression, and (C) anxiety scores among 59 transition-age youth using OTX-202 concurrently with outpatient mental health treatment. PROMIS: Patient-Reported Outcomes Measurement Information System.

Changes in SSI scores are displayed in Figure 2A. Relative to baseline, mean SSI scores significantly decreased (*F*_12,705_=9.9; *P*<.001; *d*=0.95), and the percentage of participants denying suicidal ideation significantly increased (*F*_12,705_=4.9; *P*<.001). Mean SSI scores were significantly reduced by week 2 (mean difference −2.7, 95% CI −1.2 to −4.1; *t*_705_=3.7; *P*<.001; *d*=0.54) and continued to decrease through week 12 (mean difference −5.1, 95% CI −3.7 to −6.5, *t*_705_=7.1; *P*<.001; *d*=0.95). The percentage of participants denying suicidal ideation significantly increased from 5.1% at baseline to 51.7% at week 12 (*t*_705_=5.2; *P*<.001).

### Change in Psychological Symptoms

Changes in PROMIS depression and anxiety scores are displayed in Figures 2B and 2C. Relative to baseline, mean PROMIS depression (*F*_12,705_=9.6; *P*<.001) and anxiety (*F*_12,705_=9.1; *P*<.001) scores significantly decreased, and the percentage of participants with scores within normal limits of the general population increased (for depression, *F*_12,705_=3.3; *P*<.001 and for anxiety, *F*_12,705_=1.9; *P*=.02). Mean depression and anxiety scores were significantly improved by week 1 (for depression, mean difference −2.1, 95% CI −0.2 to −3.9; *t*_705_=2.2; *P*=.03; *d*=0.33 and for anxiety, mean difference −2.8, 95% CI −0.9 to −4.8; *t*_705_=2.8; *P*=.001; *d*=0.41) and continued to improve through week 12 (for depression, mean difference −6.8, 95% CI −5.0 to −8.7; *t*_705_=7.2; *P*<.001; *d*=0.78 and for anxiety, mean difference −7.5, 95% CI −5.5 to −9.5; *t*_705_=7.4; *P*<.001; *d*=0.83). The percentage of participants with depression scores within normal limits had significantly increased from 11.9% at baseline to 32.2% by week 2 (*t*_705_=3.0; *P*=.002) and continued to increase to 40.4% at week 12 (*t*_705_=4.3; *P*<.001). The percentage of participants with anxiety scores within normal limits significantly increased from 10.2% at baseline to 24.5% at week 2 (*t*_705_=2.4; *P*=.02) and continued to increase to 34.1% at week 12 (*t*_705_=5.8; *P*<.001).

### Suicide Attempts

From baseline to week 12, a total of 3 (5.1%, 95% CI 0%-11.2%) participants reported a suicide attempt (3 actual attempts and 0 interrupted or aborted attempts).

### Device Safety

The number, severity, and nature of reported AEs are summarized in Table 2. Of 39 total AEs, none were rated as severe or as possibly or definitively related to device use. Two of the suicide attempts were classified as AEs with moderate severity, and 1 was classified as an AE with mild severity.

**Table 2:** Number of adverse event (AE) types, severity, and relatedness (n=39).

AE type	Severity rating, n (%)				Related to device, n (%)			
	Mild	Moderate	Severe	Definitely not		Unlikely	Possibly	Definitely
Suicide attempt	2 (5.1)	1 (2.6)	0 (0)	2 (5.1)		1 (2.6)	0 (0)	0 (0)
Hospital or clinic visits	3 (7.7)	2 (5.1)	0 (0)	5 (12.8)		0 (0)	0 (0)	0 (0)
For a medical condition	3 (7.7)	1 (2.6)	0 (0)	4 (10.3)		0 (0)	0 (0)	0 (0)
For suicidal ideation	0 (0)	1 (2.6)	0 (0)	1 (2.6)		0 (0)	0 (0)	0 (0)
Missed study visits	31 (79.5)	0 (0)	0 (0)	31 (79.5)		0 (0)	0 (0)	0 (0)

### Intervention Acceptability and Usability

Weekly mean SUS scores ranged from 84.2 (SD 9.2) to 85.8 (SD 9.0), placing OTX-202 at the 97th percentile of usability [29]. Of 59 participants enrolled, 56 (94.6%) completed the first session, 47 (79.7%) completed at least 6 sessions, and 24 (40.7%) completed all 12 sessions. Participants completed a mean of 9.0 (SD 3.5; range 1-12) sessions.

## Discussion

In this phase 1 clinical trial of transition-age youth engaged in outpatient mental health care, the severity of suicidal ideation significantly decreased concurrently with OTX-202 app use. Statistically significant reductions were observed within 2 weeks of baseline using both EMA and weekly self-report. By week 12, more than half of the participants reported they were no longer experiencing suicidal ideation. Participants also reported statistically significant improvements in depression and anxiety symptoms. The large improvements in suicidal ideation from baseline to week 12 (ie, *d*=0.95) were comparable in magnitude to those observed among high-risk patients receiving psychiatric treatment receiving suicide-focused CBTs in addition to usual care in multiple previous randomized clinical trials [17,18,22,23]. The suicide attempt rate in this sample (3/59, 5.1% of enrolled patients by week 12) was also comparable to previously reported suicide attempt rates among high-risk patients receiving psychiatric treatment receiving suicide-focused CBTs in addition to usual care (2.6% to 16.5% by week 12).

It is important to emphasize that this study’s single-arm design cannot establish the efficacy of OTX-202. Because participants were required to be actively engaged in mental health treatment while enrolled in this study (a design choice made to comply with FDA requirements for safety monitoring), we are unable to differentiate the app’s effects on clinical outcomes from the effects of concurrent psychotherapy, medication management, and other nonspecific factors (eg, monitoring by the study team). This limitation is not unique to this study; previous studies of suicide-focused CBTs have allowed (though not required) participants to continue receiving usual care while enrolled. Suicide-focused CBTs are thus better understood as add-on treatments rather than replacement treatments. In this study, engagement in mental health treatment was required by the FDA to facilitate safety monitoring. Although these findings do not establish efficacy, they support further testing of the app in phase 2 clinical trials using experimental designs that compare outcomes with those of usual care or another comparator. Such research could differentiate improvements in clinical outcomes related to the app from those related to other mental health treatments received concurrently with app use, as well as the potential nonspecific effects of monitoring by the study team.

This study supports OTX-202’s safety and acceptability. Regarding safety, there were no serious AEs or device-related events. Regarding acceptability, weekly SUS scores remained very high throughout the study period, placing the app at the 97th percentile of usability [29]. Therefore, participants rated OTX-202 positively and perceived the app as user-friendly. App engagement, an objective measure of acceptability, was also very high; on average, participants completed 9 of 12 modules, and 79.7% (47/59) completed at least half of the modules. Engagement rates in this study were comparable to mean session attendance during face-to-face CBT (8.9 sessions) and counseling (7.5 sessions) [24]. By comparison, a recent meta-analysis found that users of mental health digital applications often complete fewer than half of the application components [36]. Engagement in this study was also much higher than that observed in a previous clinical trial of OTX-202 conducted with psychiatric inpatients hospitalized for elevated suicide risk [17]. In that study, patients completed 4.4 modules on average, and 31% completed at least half of the modules. Differences in study population, setting, and methodology could explain this difference. In this study, participants were receiving outpatient mental health care at the time of enrollment and were encouraged to use the app during weekly visits with a researcher. By comparison, participants in the previous study were enrolled around the time of discharge from inpatient hospitalization and did not meet with anyone who supported intervention adherence. Given previous findings suggesting a dose-effect relationship between OTX-202 module completion and reductions in suicide attempt rates [17], this study implicates the potential value of formally integrating OTX-202 into mental health treatment plans.

Several limitations of this study warrant discussion. As noted previously, this study’s single-arm design precludes definitive conclusions about efficacy for reducing suicidal ideation and suicide attempts. Second, the 12-week follow-up period during this study was relatively brief, restricting our ability to examine longer-term patterns. Third, our sample was predominantly female and nonheterosexual—demographic characteristics associated with elevated rates of suicidal ideation, suicide attempts, and suicide mortality [2]. The generalizability of findings to transition-age youth who identify as male and/or heterosexual could thus be limited. Finally, because the app was not prescribed or made available by participants’ mental health care professionals, the intervention may not have been fully integrated into participants’ existing mental health care plans. Further testing of OTX-202 in a phase 2 randomized clinical trial that accounts for these issues is therefore warranted. Despite these limitations, this study supports the safety, acceptability, and potential efficacy of OTX-202, a digital therapeutic intervention designed to deliver suicide-focused CBT, for reducing suicide risk among transition-age youth engaged in outpatient mental health care.

## Data Availability

The datasets generated and analyzed during this study are not publicly available to protect participant privacy but are available from the corresponding author on reasonable request.

## Abbreviations

AE: adverse event
CBT: cognitive behavioral therapy
CONSORT: Consolidated Standards of Reporting Trials
EMA: ecological momentary assessment
FDA: Food and Drug Administration
GLIMMPSE: General Linear Mixed Model Power and Sample Size
HIPAA: Health Insurance Portability and Accountability Act
PROMIS: Patient-Reported Outcomes Measurement Information System
SITBI-R: Self-Injurious Thoughts and Behaviors Interview–Revised
SSI: Scale for Suicide Ideation
SUS: System Usability Scale

## References

[1] (2025). Suicide worldwide in 2021: global health estimates. World Health Organization.

[2] (2025). Suicide data and statistics. Centers for Disease Control and Prevention.

[3] Franklin JC, Ribeiro JD, Fox KR, Bentley KH, Kleiman EM, Huang X, Musacchio KM, Jaroszewski AC, Chang BP, Nock MK (2017). Risk factors for suicidal thoughts and behaviors: a meta-analysis of 50 years of research. Psychol Bull.

[4] (2025). Key substance use and mental health indicators in the United States: results from the 2024 National Survey on Drug Use and Health (HHS Publication No. PEP25-07-007, NSDUH Series H-60). Center for Behavioral Health Statistics and Quality, Substance Abuse and Mental Health Services Administration.

[5] van Ballegooijen W, Rawee J, Palantza C, Miguel C, Harrer M, Cristea I, de Winter R, Gilissen R, Eikelenboom M, Beekman A, Cuijpers P (2025). Suicidal ideation and suicide attempts after direct or indirect psychotherapy: a systematic review and meta-analysis. JAMA Psychiatry.

[6] Lee DJ, Bryan CJ, Rudd MD (2020). Longitudinal suicide ideation trajectories in a clinical trial of brief CBT for U.S. military personnel recently discharged from psychiatric hospitalization. Psychiatry Res.

[7] Czyz EK, King CA (2015). Longitudinal trajectories of suicidal ideation and subsequent suicide attempts among adolescent inpatients. J Clin Child Adolesc Psychol.

[8] Prinstein MJ, Nock MK, Simon V, Aikins JW, Cheah CS, Spirito A (2008). Longitudinal trajectories and predictors of adolescent suicidal ideation and attempts following inpatient hospitalization. J Consult Clin Psychol.

[9] Bryan CJ, Bryan AO, Khazem LR, Aase DM, Moreno JL, Ammendola E, Bauder CR, Hiser J, Daruwala SE, Baker JC (2024). Crisis response planning rapidly reduces suicidal ideation among U.S. military veterans receiving massed cognitive processing therapy for PTSD. J Anxiety Disord.

[10] Jobes D, Barnett J (2025). Evidence-based care for suicidality as an ethical and professional imperative: how to decrease suicidal suffering and save lives. Am Psychol.

[11] Green JD, Kearns JC, Rosen RC, Keane TM, Marx BP (2018). Evaluating the effectiveness of safety plans for military veterans: do safety plans tailored to veteran characteristics decrease suicide risk?. Behav Ther.

[12] Gamarra JM, Luciano MT, Gradus JL, Wiltsey Stirman S (2015). Assessing variability and implementation fidelity of suicide prevention safety planning in a regional VA healthcare system. Crisis.

[13] Rozek DC, Tyler H, Fina BA, Baker SN, Moring JC, Smith NB, Baker JC, Bryan AO, Bryan CJ, Dondanville KA (2023). Suicide intervention practices: what is being used by mental health clinicians and mental health allies?. Arch Suicide Res.

[14] Grumet JG, Jobes DA (2024). Zero suicide - what about "treat"?. Crisis.

[15] Goldsack J, Coder M, Fitzgerald C, Navar-Mattingly N, Corvos A, Atreja A (2019). Digital health, digital medicine, digital therapeutics (DTx): what’s the difference?. Digital Medicine Society.

[16] d'Halluin A, Costa M, Morgiève M, Sebbane D (2023). Attitudes of children, adolescents, and their parents toward digital health interventions: scoping review. J Med Internet Res.

[17] Bryan CJ, Simon P, Wilkinson ST, Allen MH, Perez J, Adler C, Moon K, Astorino L, Carpenter KM, Misquitta L, Brownlowe K, Khazem LR, Hay J, Starkey AG, Tartaglia J, Winston H, Simpson S, Dager AD, Feuerstein S (2025). A digital therapeutic intervention for inpatients with elevated suicide risk: a randomized clinical trial. JAMA Netw Open.

[18] Rudd MD, Bryan CJ, Wertenberger EG, Peterson AL, Young-McCaughan S, Mintz J, Williams SR, Arne KA, Breitbach J, Delano K, Wilkinson E, Bruce TO (2015). Brief cognitive-behavioral therapy effects on post-treatment suicide attempts in a military sample: results of a randomized clinical trial with 2-year follow-up. Am J Psychiatry.

[19] Sinyor M, Williams M, Mitchell R, Zaheer R, Bryan CJ, Schaffer A, Westreich N, Ellis J, Goldstein BI, Cheung AH, Selchen S, Kiss A, Tien H (2020). Cognitive behavioral therapy for suicide prevention in youth admitted to hospital following an episode of self-harm: a pilot randomized controlled trial. J Affect Disord.

[20] Bryan CJ, Rudd MD (2018). Brief Cognitive-Behavioral Therapy for Suicide Prevention.

[21] Brown GK, Ten Have T, Henriques GR, Xie SX, Hollander JE, Beck AT (2005). Cognitive therapy for the prevention of suicide attempts: a randomized controlled trial. JAMA.

[22] Baker JC, Starkey A, Ammendola E, Bauder CR, Daruwala SE, Hiser J, Khazem LR, Rademacher K, Hay J, Bryan AO, Bryan CJ (2024). Telehealth brief cognitive behavioral therapy for suicide prevention: a randomized clinical trial. JAMA Netw Open.

[23] Bryan CJ, Khazem LR, Baker JC, Brown LA, Taylor DJ, Pruiksma KE, Acierno R, Larick JG, Baucom BR, Garland EL, Rudd MD (2025). Brief cognitive behavioral therapy for suicidal military personnel and veterans: the Military Suicide Prevention Intervention Research (MSPIRE) randomized clinical trial. JAMA Psychiatry.

[24] Fox KR, Harris JA, Wang SB, Millner AJ, Deming CA, Nock MK (2020). Self-injurious thoughts and behaviors interview-revised: development, reliability, and validity. Psychol Assess.

[25] Beck AT, Kovacs M, Weissman A (1979). Assessment of suicidal intention: the Scale for Suicide Ideation. J Consult Clin Psychol.

[26] Broderick JE, Schneider S, Junghaenel DU, Schwartz JE, Stone AA (2013). Validity and reliability of patient-reported outcomes measurement information system instruments in osteoarthritis. Arthritis Care Res (Hoboken).

[27] Lewis JR (2018). The System Usability Scale: past, present, and future. Int J Hum Comput Interact.

[28] Kortum PT, Bangor A (2013). Usability ratings for everyday products measured with the System Usability Scale. Int J Hum Comput Interact.

[29] Sauro J (2011). A Practical Guide to the System Usability Scale: Background, Benchmarks and Best Practices.

[30] Levine J, Schooler NR (1986). SAFTEE: a technique for the systematic assessment of side effects in clinical trials. Psychopharmacol Bull.

[31] 21 CFR part 11 - electronic records; electronic signatures. Code of Federal Regulations.

[32] Eysenbach G, CONSORT-EHEALTH Group (2011). CONSORT-EHEALTH: improving and standardizing evaluation reports of web-based and mobile health interventions. J Med Internet Res.

[33] Keselman HJ, Algina J, Kowalchuk RK, Wolfinger RD (2010). A comparison of two approaches for selecting covariance structures in the analysis of repeated measurements. Commun Stat Simul Comput.

[34] Power curves for multi-level studies. Kleiman Lab.

[35] Kreidler SM, Muller KE, Grunwald GK, Ringham BM, Coker-Dukowitz ZT, Sakhadeo UR, Barón AE, Glueck DH (2013). GLIMMPSE: online power computation for linear models with and without a baseline covariate. J Stat Softw.

[36] Garrido S, Millington C, Cheers D, Boydell K, Schubert E, Meade T, Nguyen QV (2019). What works and what doesn't work? A systematic review of digital mental health interventions for depression and anxiety in young people. Front Psychiatry.

